# Effects of straw application on nitrate leaching in fields in the Yellow River irrigation zone of Ningxia, China

**DOI:** 10.1038/s41598-017-18152-w

**Published:** 2018-01-17

**Authors:** Shiqi Yang, Yongsheng Wang, Ruliang Liu, Quanxin Li, Zhengli Yang

**Affiliations:** 1 Institute of Environment and Sustainable Development in Agriculture, Chinese Academy of Agricultural Sciences, Beijing, 100081 China; 2 Key Laboratory of Agro-Environment and Climate Change, Ministry of Agriculture, Beijing, 100081 China; 3 Key Laboratory of Regional Sustainable Development Modeling, Institute of Geographic Sciences and Natural Resources Research, Chinese Academy of Sciences, Beijing, 100101 China; 4 Institute of Agricultural Resources and Environment, Ningxia Academy of Agro-Forestry Science, Yinchuan, 750002 China; 5 Institute of Agricultural Resources and Regional Planning, Chinese Academy of Agricultural Sciences, Beijing, 100081 China

## Abstract

A five-year field experiment was conducted to investigate the effects of straw application on nitrate leaching loss. Treatments included soil that was not treated (control), soil treated with straw at a low rate (4,500 kg ha^−2^, T1) and soil treated with straw at a high rate (9,000 kg ha^−2^, T2). Nitrate-nitrogen leaching in the 10, 20, 30, 60, and 90 cm soil layers was measured using the resin-core method. The results indicated that straw application could reduce soil nitrate leaching losses in the 0–30 cm layer. In this layer, the nitrate leaching values for T1 (13.76 kg ha^−2^) and T2 (13.74 kg ha^−2^) were both significantly lower than those of the control (15.76 kg ha^−2^) (P < 0.05); the soil nitrate leaching losses decreased by 12.71% and 12.84% for those two treatments, respectively. However, no significant differences in losses were observed (P > 0.05) between T1 and T2. The effects of straw application were apparent only in the ploughing layer (30 cm-depth soil layer). In the deeper layers (60 and 90 cm), no significant differences were observed between the treatments and the control, and the same results were observed in the topsoil layers (10 and 20 cm).

## Introduction

Agricultural nonpoint source pollution has dramatically impacted water quality in China. The total nitrogen (TN), total phosphorus (TP) and chemical oxygen demand (COD) from agricultural sources are approximately 2.7, 0.28, and 13.24 million tons per year, respectively, accounting for 57.2%, 67.4%, and 3.7% of total emissions, respectively^1^. For the top ten rivers in China, 10.2% are considered inferior class V, and 20.9% are class IV-V. In China, 24 of 61 key lakes and pools have a water quality below class III, and nearly 0.3 billion people in rural regions drink unsafe water^2^. The Yellow River irrigation zone of Ningxia is the 4^th^ largest irrigation zone in China by area, yet it is a black box in terms of water pollution because of its nonpoint source pollution. Ammonium-nitrogen concentrations in drainage channels are often 20–30 mg L^−1^ (maximal concentration of 70 mg L^−1^). Only 38.3% of the water tested meets the Chinese national standard, and many of the samples are below class V. The water quality of the Yellow River in Ningxia is usually class V all year round. In the tenth Five-Year Plan for China, the acceptable level for ammonium-nitrogen concentrations in the water was increased from 2.57 to 3.94 mg L^−1^, yet only 38.3% of surface water met the national standard during the eleventh Five-Year Plan of China. The nitrate concentration of almost half of the shallow groundwater in China exceeded 10 mg L^−1^ ^3^. Similar problems occur in other irrigation zones in China. More than half of the groundwater in 14 investigated counties in the North China Plain have nitrate concentrations that exceed 10 mg L^−1^ ^4^. The nitrate concentration in 7.4% of the groundwater in Beijing suburbs exceeds the national standard for groundwater^5^.

Many studies have addressed the relationship between organic matter (OM) applications and soil nitrate losses. Some scientists believe that OM can reduce nitrate losses^6,7^, but excess straw can result in increased nitrate leaching^8–10^. OM compost can reduce mineralization rates and nitrate losses^11,12^. OM practices typically aim to maintain a high carbon/nitrogen ratio and are used to reduce soil soluble nitrogen release in a short time frame. In Europe, OM applications are limited to 110–140 kg ha^−2^ per season; these rates can markedly decrease nitrate losses^13^. Manures replace some chemical fertilizers and can help control soil nitrogen release^14^. Losses from annual nitrate leaching can be 4.4–5.6 times greater in conventional plots than in organic plots as well as in integrated plots located between conventional and organic plots. Therefore, compared with conventional practices, organic and integrated fertilization practices more actively and efficiently support denitrifier communities, shift the balance of nitrogen emissions and nitrate losses, and cause less environmental harm^15^. Nitrates mainly originate from the mineralization of nonleguminous soil organic matter (SOM); only one-quarter of nitrates originate from leguminous SOM^16^. OM applications can reduce inorganic nitrate leaching, and wheat straw is better than either sawdust or lignite powder^17^. Many studies have revealed environmental advantages of compost regarding reducing both mineralization rates and the potential for nitrate leaching and delaying the change of organic nitrogen to nitrate^18^. Mamo determined that nitrate leaching could be reduced by substituting compost for commercial inorganic fertilizer in vegetable fields^19^. Brinton reported lower potential nitrate leaching from soils amended with compost rather than uncomposted manure in a corn field^20^. The application of rice straw compost can reduce nitrate concentrations in test plots, which implies that the incorporation of rice straw reduces the nitrate concentration not only in surface water but also in percolated water at a depth of 10 cm; the nitrate concentration was much lower at a depth of 20 cm than at 10 cm in the test plots^21^. In another study, although only approximately 10% of the applied ^15^N-Iabelled fertilizer remained in the 0–30 cm layer of the control and plant manure plots, more than 25% of the applied ^15^N remained in the pig compost plot^22^. An inverse relationship between dissolved organic carbon (DOC) and nitrate concentrations in streams in Japan is closely related to excess nitrogen availability together with a carbon deficit in the soil environment^23^. In a multi-site, four-year study, the application of organic materials to soils enhanced the immobilization of nitrogen into microbial biomass and therefore reduced inorganic nitrogen concentrations at all test sites in all 4 years of the study^24^. If the goal is to reduce nitrate losses, organic fertilizer application rates should not exceed 175 kg N ha^−2^ in nitrate-vulnerable zones. However, at the Rothamsted Experimental Station, the index was 276 kg N ha^−2^ ^25^. Bird guano application resulted in more nitrate losses when the application amount exceeded 11.2 × 10^3^ kg ha^−2^ ^26^.

In the Ningxia irrigation zone, the SOM is usually deficient; the values range from 9.2 to 14.5 g kg^−1^, and the average value is 10.2 g kg^−1^. Nitrogen fertilizer application rates can reach 301 kg ha^−2^, which is 1.6 times greater than the national average. In the surface water in this region, 61–66% of the TN and 76–81% of the nitrite originates from farmland sources. Approximately 7 billion m^3^ y^−1^ of water is drawn from the Yellow River, and 2.5 billion m^3^ y^−1^ enters the Yellow River again by percolation and filtration from upland fields. The average irrigation rates are 15–45 × 10^3^ m^3^ ha^−2^ for rice, 3.3–4.5 × 10^3^ m^3^ ha^−2^ for wheat/corn (intercropping), and 1.5 × 10^3^ m^3^ for winter irrigation and spring irrigation ha^−2^ ^27^. These data suggest that the Ningxia irrigation zone is a major region of nonpoint source pollution. The objective of the present field experiments was to prove that straw application affects the control of water pollution, and the long-term goal is to extensively improve straw application in China, especially within irrigation zones.

## Materials and Methods

### Study zone

**Figure 1:**
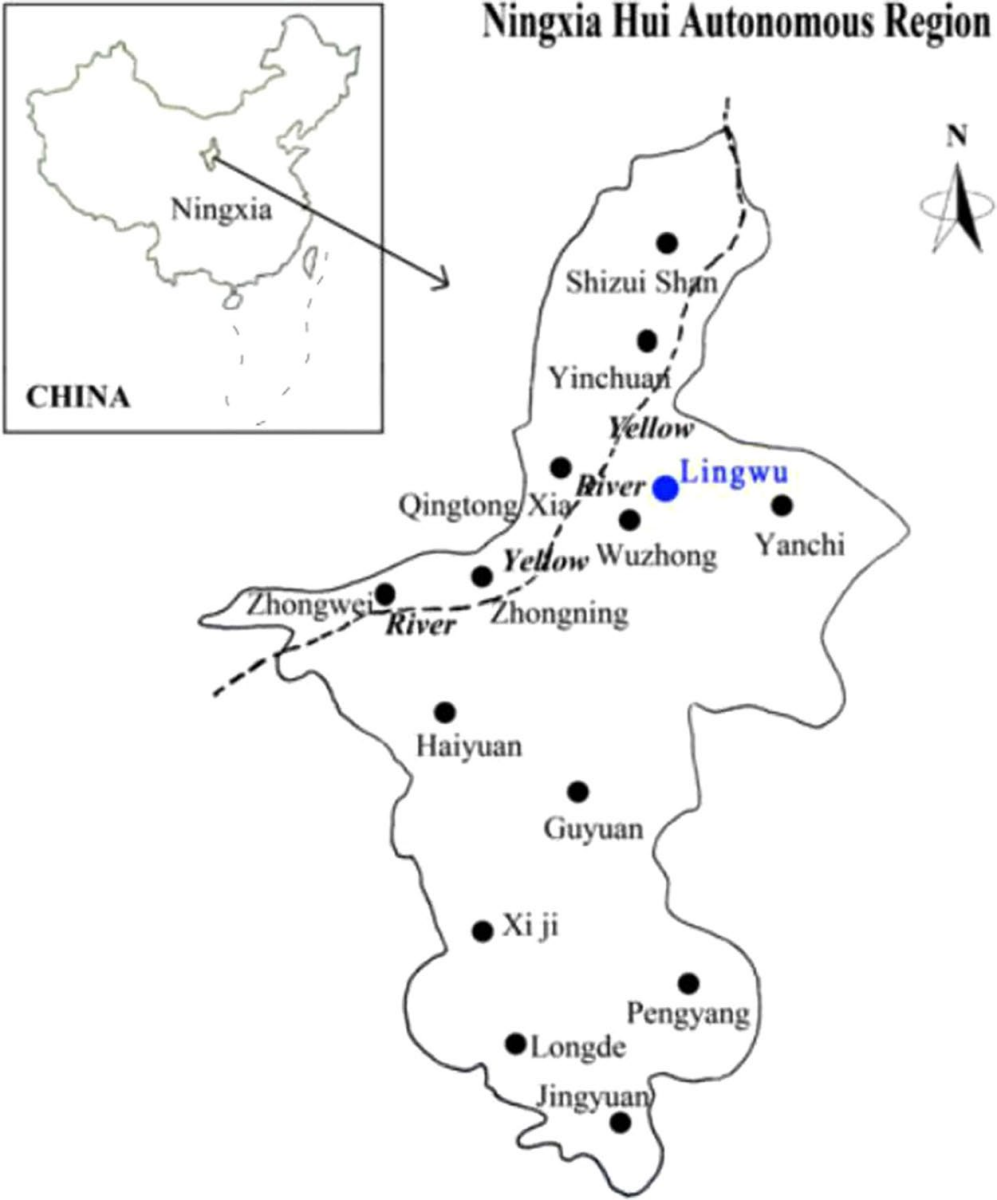
Location of the study zone in North Ningxia. (Note: The figure has been published in Shiqi Yang *et al*. Effect of Nitrate Leaching Caused by Swine Manure Application in Fields of the Yellow River Irrigation Zone of Ningxia, China *Scientific Reports*. | 7: 13693 | 10.1038/s41598-017-12953-9, https://www.nature.com/articles/s41598-017-12953-9.pdf).

The field trial (106°17′52″E, 38°07′26″N) was located in Lingwu County in the Ningxia Autonomous Region. Tvhis area is part of the upstream irrigation zone of the Yellow River (Fig. 1) and is in a temperate arid zone, which consists of low rainfall and a dry climate. The rainy season is from July to September and accounts for 70% of the total annual precipitation. Snow seldom occurs in winter. The average annual precipitation is only 193 mm y^−1^, and the evaporation rate is 1,763 mm y^−1^. There are only approximately 150–164 frost-free days. The average annual temperature is 8.9 °C, and the active accumulated temperature ≥10 °C ranges from 3,200–3,400 °C. The average elevation above sea level is 1,130 m, and there are between 2,800 and 3,100 annual daylight hours in this area. The water used for agriculture in the area relies on gravity flow systems. The soil type is classified as anthropogenic-alluvial with low fertility (Table 1). The topsoil is highly saline and has a high pH, and the area is in a single-harvest zone. The main crops are rice, corn, and spring wheat, and the typical cropping system is a rice-corn rotation.

**Table 1:** Physical and chemical properties of the field in this study.

Soil depth (cm)	Bulk density (g cm^−3^)	SOM (g kg^−1^)	TN (g kg^−1^)	TP (g kg^−1^)	Soil available nutrients (mg kg^−1^)					Soil salt %	pH
					N	P	K	NO_3_^−^-N	NH_4_^+^-N		
00–30	1.49	13.78	0.87	0.80	93.43	27.34	145.87	10.21	0.98	1.13	8.59
30–60	1.58	7.33	0.75	0.78	63.76	14.89	137.10	6.97	0.96	0.91	8.76
60–90	1.43	4.14	0.49	0.50	34.17	4.42	98.25	6.23	0.93	0.74	9.21

### Experimental design

Each plot was insulated by brick walls lined with plastic film to prevent water exchange; these walls were 40 cm high above the ground and 80 cm deep beneath the soil. There were 3 treatments: no straw application (CK: 0 kg ha^−2^), semi-straw (SS) application (T1: 4,500 kg ha^−2^), and total straw (TS) application (T2: 9,000 kg ha^−2^). The plot area was 200 m^2^ and was replicated 3 times. The field trials were conducted from 2009 to 2013. The crop was rice each year, with the exception of 2011, during which the crop was winter wheat. The straw was manually cut to 10 cm pieces and then ploughed into 0–25 cm of the topsoil before winter irrigation.

The fertilizer application rates in the rice production field were as follows: urea, 300 kg N ha^−2^; triple superphosphate, 105 kg P_2_O_5_ ha^−2^; and potassium chloride, 60 kg K_2_O ha^−2^. Half of the nitrogen and all of the total phosphate and potash were applied as basic fertilizers, and the other 50% of the nitrogen was applied as a topdressing at the seeding (end of May), tillering (end of June), and booting (end of July) stages at a proportion 3:1:1. The time of the first irrigation in the field was approximately mid-May each year, and the last irrigation occurred in early July of each year. The irrigation rate was 15,000 m^3^ ha^−2^ during the rice growing period. Rice transplanting was conducted in mid-May, and harvesting occurred in late September (approximately 120 days).

The fertilizer application rates in the wheat field were as follows: urea, 225 kg N ha^−2^; triple superphosphate, 150 P_2_0_5_ kg ha^−2^; and potassium chloride, 90 kg K_2_O ha^−2^. Half of the nitrogen and all of the phosphate and potash were applied as basic fertilizers. The remaining 50% the nitrogen was applied as a top dressing at a ratio of 3:1:1 at the seeding (early March), elongation (early May), and earing (early June) stages. The irrigation scheme was as follows: winter irrigation, 1,350 m^3^ ha^−2^ (end of October); greening, 900 m^3^ ha^−2^ (end of March); elongation, 1,050 m^3^ ha^−2^ (mid-May); and heading, 1,050 m^3^ ha^−2^ (early June). Sowing occurred on 4 Oct. 2010, and harvest occurred on 29 Jun. 2011 (269 days).

### Methods

**Figure 2:**
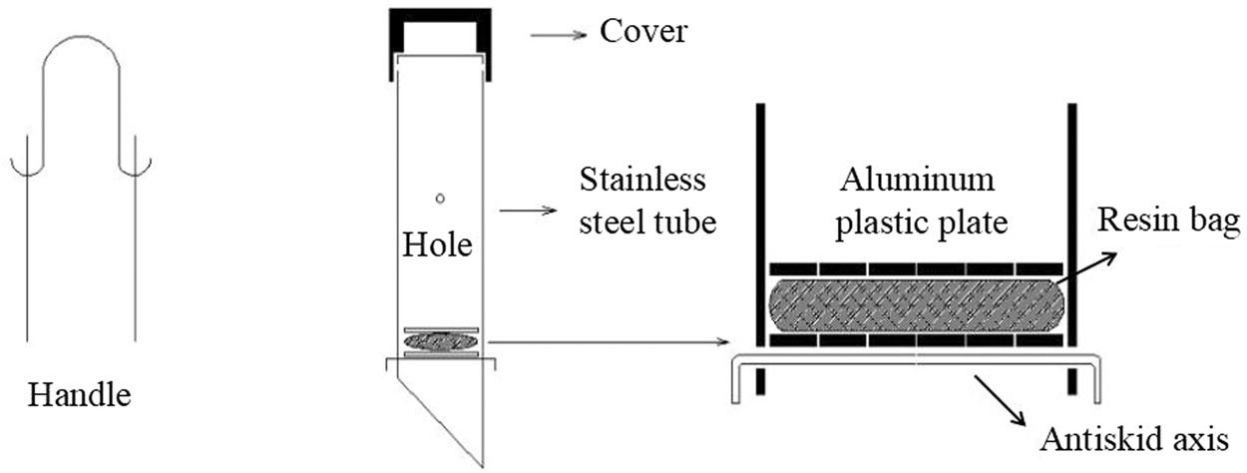
Resin-core device used to measure soil nitrate loss.

Soil nitrate losses were determined using a resin-core device (Fig. 2, *China National Patent No*. *201020282864*.*4*, 2010). This method has been used previously to determine soil nitrogen mineralization^12,28,29^. The resin-core device is composed of a stainless steel pipe (76 mm diameter × 0.82 mm thickness), a resin bag (60-mesh nylon net, 8 cm long × 8 cm wide), 2 pieces of an aluminium plastic material plate (74 mm diameter with 13 eyes (3 mm diameter)), a cover, a handle and an antiskid axis. The resin used was a strongly alkaline anion exchange resin (trademark #717), and the pretreatment was in accordance with the method for pretreating ion exchange resins (*GB/T* 5476-1996). There were 4 holes in the pipe body for reducing the pressure differential inside and outside of the pipe.

The end of the pipe was a 10 cm-long wedge surface for easy insertion into the soil. A 2 cm-long aluminium plastic plate was above the wedge. The antiskid axis can be fixed to prevent the aluminium plastic plates from dropping. The cover and the handle were convenient for placing and inserting the pipe body in the soil. There were 5 types of resin-core devices; the types were 22, 32, 42, 72, and 102 cm long. Initially, the resin-core devices were inserted just below the soil surface. The pipes were later removed, and the soil in the wedge and 2 cm above the wedge was collected. However, the aluminium plastic plates, resin bags (16 g of resin per bag), aluminium plastic plates, and antiskid axis were later attached in sequential order. The devices were ultimately retrieved, the old resin bags were removed, and new resin bags were installed for the next test stage. The old resin bags were subsequently refrigerated. Three types of devices were placed in each plot in a line and separated by 2 m, and 3 replications were situated along the diagonal of each plot.

The nitrate in the resin bags was extracted with potassium solution (1 mol L^−1^ KCl), and measured using ultraviolet spectrophotometry. The nitrate loss was calculated using the following equation:

The data were analysed using SPSS 19 and Excel 2010. One-way analysis of variance (ANOVA) was used to test for significant (α = 0.05) differences among the different treatments.

## Results

### Nitrate leaching losses

**Figure 3:**
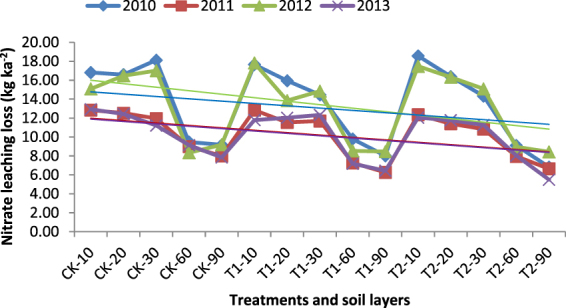
Nitrate leaching trends for the 4 years.

Figure 3 shows the trends of nitrate leaching. Nitrate leaching losses in each treatment decreased as the soil depth increased. Additionally, T1 lost more than the control did, and T2 lost more than T1 did. Nitrate leaching losses in the rice field (2010, 2012, and 2013) were greater than the losses in the winter wheat field (2011). The trends for each of the 3 years in the rice fields were very similar. These results indicated that straw application was beneficial for reducing nitrate leaching.

Table 2 shows the nitrate leaching losses. Straw application reduced nitrate leaching losses in the 30 cm layer. The treatment groups and the control group significantly differed (P < 0.05), with the exception that no obvious differences between T1 and T2 in 2011 (P > 0.05) were observed. The nitrate leaching losses ranged from 14.06–18.12 kg ha^−2^ in the rice field and from 10.83–11.95 kg ha^−2^ in the winter wheat field. Above the 30 cm layer, no significant differences were observed between the treatment groups. Below the 30 cm layer, no significant differences (P > 0.05) were observed among T1, T2, and the CK. The results for the 4 years show that straw application could reduce soil nitrate leaching losses in the 30 cm layer, the losses in both T1 (13.76 kg ha^−2^) and T2 (13.74 kg ha^−2^) significantly differed from those in the control (15.76 kg ha^−2^) (P < 0.05), and soil nitrate leaching losses decreased 12.71% and 12.84% in T1 and T2, respectively.

**Table 2:** Nitrate leaching losses at different layers and in different years.

Soil layers	Treatments	Nitrate leaching losses (kg ha^−2^)					Mean of the same soil layer
		2010	2011	2012	2013	Mean of treatments	
10 cm	CK	16.81a	12.85a	15.08b	14.39b	14.78a	15.83a
	T1	17.65a	12.83a	17.81a	18.17a	16.62a	
	T2	18.57a	12.36a	17.47a	16.00a	16.10a	
	Mean	17.68a	12.68b	16.79a	16.19a	—	
20 cm	CK	16.59a	12.52a	16.47a	16.40a	15.49a	15.20a
	T1	15.94a	11.54a	13.84a	18.34a	14.92a	
	T2	16.38a	11.40a	16.31a	16.65a	15.18a	
	Mean	16.30a	11.82b	15.54a	17.13a	—	
30 cm	CK	18.12a	11.95a	17.01a	15.98a	15.76a	14.42b
	T1	14.45b	11.69ab	14.84b	14.06b	13.76b	
	T2	14.33b	10.83b	15.08b	14.71b	13.74b	
	Mean	15.63a	11.49b	15.64a	14.92a	—	
60 cm	CK	9.47a	9.00a	8.34a	8.20a	8.75a	8.59c
	T1	9.79a	7.26a	8.53a	8.30a	8.47a	
	T2	9.11a	7.96a	8.96a	8.12a	8.54a	
	Mean	9.46a	8.07b	8.61ab	8.21b	—	
90 cm	CK	9.17a	8.00a	9.19a	8.65a	8.75a	7.99c
	T1	8.01a	6.26a	8.46a	7.98a	7.68a	
	T2	6.82a	6.65a	8.43a	8.22a	7.53a	
	Mean	8.00ab	6.97b	8.69a	8.29ab	—	
Mean of 5 soil layers		13.41a	10.21b	13.06a	12.95a	—	12.41

Notes: α = 0.05, a and b stand for significant differences with respect to nitrate leaching losses.

### Characteristics of soil nitrate leaching losses

#### Soil nitrate leaching losses and crops

The results (Table 2) for rice (13.41, 13.06, and 12.95 kg ha^−2^) and winter wheat (10.21 kg ha^−2^) showed that the soil profiles in the fields significantly differed (P < 0.05); the nitrate leaching losses were more substantial in the rice field than in the winter wheat field. If the goal for the region is to reduce nitrate leaching, then the area devoted to rice production should be reduced. No significant differences (P > 0.05) were observed between years (2010, 2011, and 2013).

#### Soil nitrate leaching losses and soil layers

Significant differences were observed in nitrate leaching losses (P < 0.05) between the topsoil layers (15.83 kg ha^−2^ for the 10 cm layer and 15.20 kg ha^−2^ for the 20 cm layer), the 30 cm layer (14.42 kg ha^−2^) and the deep layers (8.59 kg ha^−2^ for the 60 cm layer and 7.99 kg ha^−2^ for the 90 cm layer). No significant differences (P > 0.05) were observed between the 10 cm and 20 cm layers or between the 60 cm and 90 cm layers. Straw mainly stayed within the top 20 cm of soil; no significant differences were observed between the layers up to 20 cm. Nitrate leaching losses in the 60 cm and 90 cm layers were not impacted by straw applications because of the short duration of the trial period. Therefore, the critical layer for nitrate leaching was the 30 cm layer, which was also the bottom of the soil ploughing layer.

#### Process of soil nitrate leaching loss

**Figure 4:**
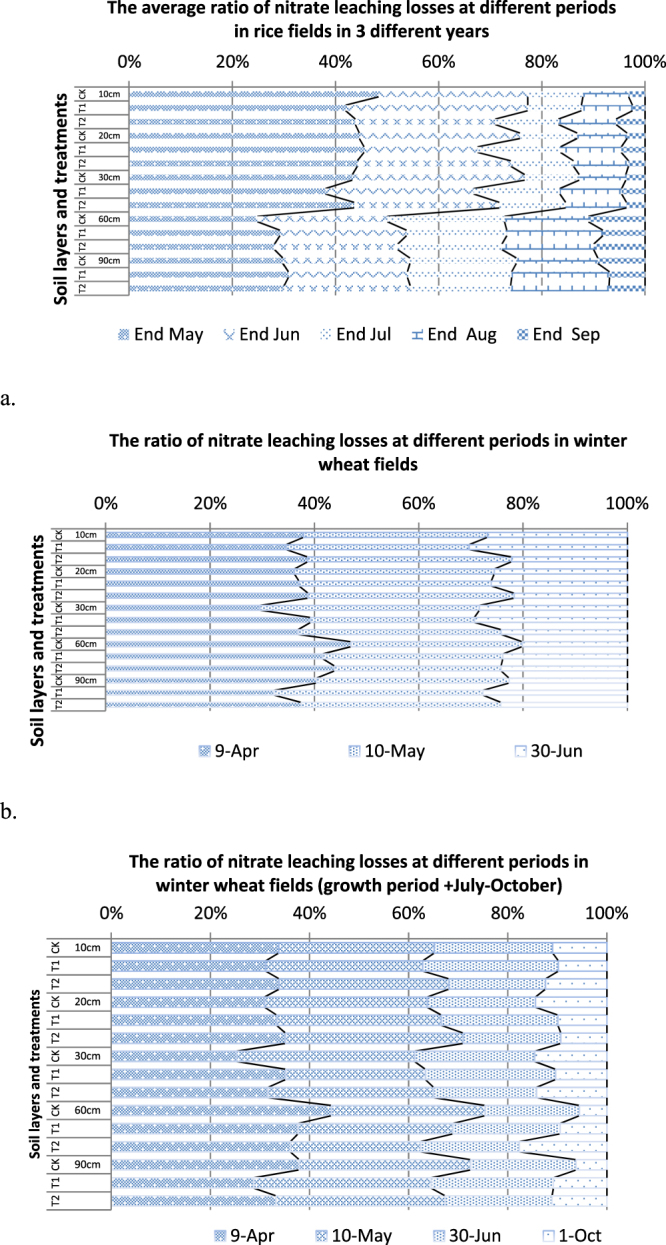
Ratios of nitrate leaching losses during the entire growth period.

Figure 4 shows the ratios of nitrate leaching losses for different periods. In the rice field, the key period of soil nitrate leaching occurred before the end of June (45–50 days/130–140 growing days) (Fig. 4a.); nearly 80% of the nitrate leached above the 30 cm layer, and almost 50–55% of the nitrate leached below the 30 cm layer. In the winter wheat fields, nearly 80% (Fig. 4b) of the nitrate leaching losses from each soil layer occurred before early June, and almost 70% (Fig. 4c) of the nitrate leaching losses from each aggregate layer occurred during the four months prior to October. The key periods of soil nitrate leaching losses occurred during the early growth stages of rice and winter wheat; for rice, the key period occurred at end of June (tillering phase). Another key period for winter wheat occurred at the beginning of June (earing phase).

## Discussion

### Soil nitrate concentration

**Figure 5:**
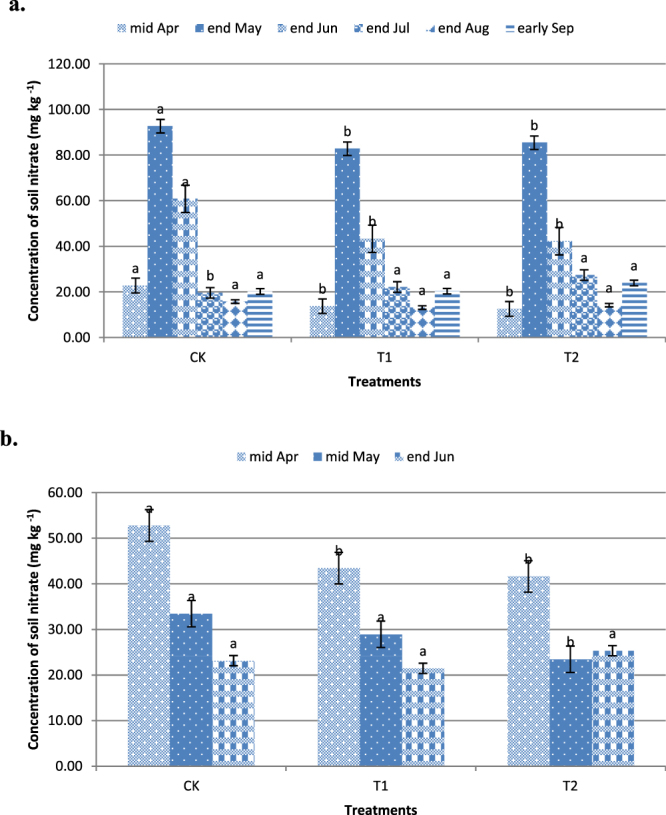
Soil nitrate concentrations in the 0–30 cm soil layer.

The soil nitrate concentration is the key factor influencing nitrate leaching losses. Figure 5 shows the course of changes in the soil nitrate concentration in the 0–30 cm layer at different time points. In the rice field, the annual mean of the soil nitrate concentration was clearly higher (P < 0.05) in May and June than in other months (Fig. 5a). In the winter wheat field, the most critical period of leaching was during April (Fig. 5b), and the nitrate concentration was much higher during this time than during the other two months of measurements (P < 0.05). Not surprisingly, the periods of high nitrate concentrations were also periods of high nitrate leaching. The nitrate concentration in the treatments was lower than that in the control treatments in the rice field. Compared with that in the control treatment, the nitrate concentration in the T1 and T2 treatments both significantly differed (P < 0.05) in April, May, June, and July; no significant differences were observed between the controls and the treatments in August or September. However, significant differences were observed between the T1 and T2 treatments at all time points (P > 0.05). In the winter wheat field, the nitrate concentrations in the treatments and the controls significantly differed in April. However, the control treatment and the T1 treatment were not significantly different in May, but the T1 and T2 treatments were significantly different in May. The results show that straw application was beneficial for reducing the soil nitrate concentrations during the early stages of rice growth. However, in the middle and at the end of the rice growing stage, the soil nitrate leaching did not increase despite the soil nitrate concentration increasing slightly. Although the straw application constituted an additional source of nitrogen, the nitrate leaching losses did not increase. Wang demonstrated that nitrate and nitrite concentrations clearly decrease after straw applications to paddy fields and that these concentrations are negatively related to straw application rates^30^. The processes of soil nitrogen mineralization and fixation occur simultaneously; more fixation occurs relative to mineralization during the early stages of straw decomposition^31^. A 16-year-long study of corn straw applications involved measurements of nitrate accumulations in the 0–200 cm soil layer in a semi-arid region in North China^32^.

### Nitrate in the soil leachate

**Figure 6:**
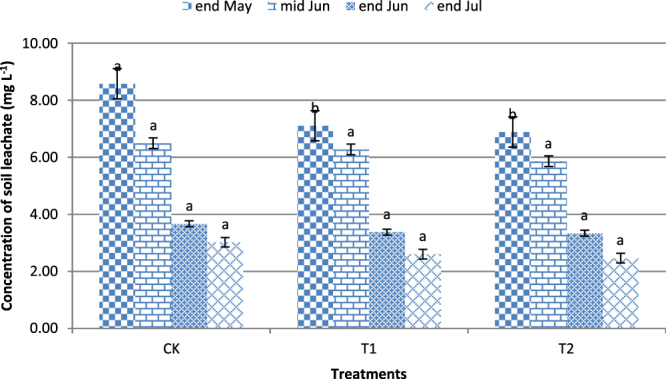
Nitrate concentrations in soil leachate of the 30 cm layer.

Figure 6 shows the change in nitrate concentrations in the soil leachate of the 30 cm layer in rice fields.

The nitrate concentrations in the soil leachate were higher in May and mid-June than at the end of June and the end of July, and there was a decreasing trend of nitrate concentration over time. The nitrate concentrations in T1 and T2 did not significantly differ (P > 0.05) from those in the control, with the exception of the concentrations in May. Gollany determined that straw applications could clearly reduce the nitrate concentrations in soil leachates and could reduce nitrate leaching losses^33–35^. Straw application can reduce the nitrate and nitrite concentrations in surface water and in percolating water in paddy fields because straw enhances crop nitrogen absorption; thus, inorganic nitrogen concentrations would decrease, and nitrogen leaching losses would also decrease^36^. Keeney noted that straw applications increase soil organic carbon and lead to soil fertilizer nitrogen fixation^37^. Diao reported that straw applications can reduce the nitrate concentration in the soil leachate at the final stage of the rice growing period; the nitrate concentrations were similar to those in the control in the early stages of the rice growing period, but the nitrite concentration of the soil leachate improved^38^. A reason for the improved nitrite concentration was that nitrate was fixed by the straw^39^, while organic acids from the straw decomposition restricted the nitrite, enabling its conversion to nitrate^40^. However, Zhang reported that straw application increases soil inorganic nitrogen leaching losses^41^. Straw applications may also have increased nitrate losses in rice fields in South China^42^.

### Soil TN changes

Soil TN was improved by straw application (determined in 2013). The TN values in the control, T1, and T2 treatments were 0.86, 0.89, and 0.90 g kg^−1^, respectively, in the 0–10 cm layer; compared with the control values, the T1 and T2 values improved by 3.25% and 4.31%, respectively. In the 0–20 cm layer, the TN values in the control, T1, and T2 treatments were 0.78, 0.82, and 0.89 g kg^−1^, respectively; compared with the control values, the T1 and T2 values improved by 4.83% and 13.89%, respectively. In the 0–30 cm layer, the TN values of the control, T1, and T2 were 0.64, 0.66, and 0.69 g kg^−1^, respectively; compared with the control values, the T1 and T2 values improved by 2.76% and 6.83%, respectively. The TN improved by 0.5 g kg^−1^ in response to 8 years of continuous applications of wheat and corn straw in North China^43^. Straw application clearly increased the TN and SOM in a single-cropping rice system and in a double-cropping rice system^44^. Organic nitrogen is the key form of soil nitrogen; inorganic nitrogen is only 1% of the soil TN. Organic nitrogen can be absorbed by crops under conditions of soil microbial decomposition and mineralization^45^ and is a kind of natural controlled-release fertilizer that is controlled by soil microbes. The carbon/nitrogen ratio of straw is usually greater than that of soil microbes, so soil microbes can absorb inorganic nitrogen and convert it to microbial nitrogen^46^. Furthermore, soil inorganic nitrogen concentrations decrease, and nitrate losses are reduced.

### SOM

Compared with that in the CK (13.78 g kg^−1^), the SOM in T1 and T2 increased by 0.89 g kg^−1^ and 1.24 g kg^−1^, respectively, in the 0–30 cm layer at the end of September in 2013.

The decomposition of straw consumes soil inorganic nitrogen, decreases the accumulation of alkali-hydrolysable nitrogen in the short term and reduces the risk of nitrogen leaching. SOM contributes to improved soil water-stable aggregates and absorbs more nitrate and nitrite^47,48^; in the present study, the macroaggregates in T1 and T2 increased by 7.4% and 12.8%. Straw application can reasonably maintain the soil carbon/nitrogen ratio and reduce soil nitrogen leaching losses^49^. Straw application can enhance nitrogen supplies and reduce fertilization rates, nitrogen losses, and nitrogen pollution problems in agricultural ecosystems^50^. In Northeast China, straw application can improve the SOM and TN and benefit the formation of macroaggregates, which can adsorb soil nutrients and reduce losses^51^. The SOM was shown to increase by 4.9 g kg^−1^ when wheat and corn straw was applied continuously for 8 years^43^. Straw application can supply nitrogen to the soil, reducing fertilizer nitrogen rates, so nitrogen pollution of agricultural ecosystems could be somewhat relieved^50^. Straw application can maintain the balance between soil carbon and nitrogen, reduce soil nitrogen leaching, and improve soil structure, especially in high-fertilizer-input fields. Straw application has not reached 50% in China; there is a large gap that contrasts with developed countries.

### Cropping patterns

Soil nitrogen leaching losses were affected by cropping patterns, including crops and cropping systems. Nitrate leaching losses were greater in the rice field than in the winter wheat field; the primary reason was the presence and quantity of irrigation water. Nitrate leaching losses increased as irrigation increased. More nitrate leaching losses occurred at the seedling stage because of the small roots; soil nitrate could not be absorbed rapidly and completely at this stage. To alleviate these losses, a new fertilization technique named “the technique of backward nitrogen fertilizer” was developed; in this technique, the fertilizer rate is reduced at the early stage but shifted to the middle stage, so that the total dose is not changed^52^. The top 4 of 12 cropping patterns, which include winter wheat-rice, spring wheat-Chinese cabbage, winter wheat-silage corn and winter wheat-oil sunflower, can clearly reduce residual soil nitrate levels, so multiple cropping and intercropping systems could reduce the nitrate leaching losses and protect shallow groundwater^53^. Traditional cropping systems consist of a long fallow period from early October to early March or mid-May (150 or 220 days, respectively), and the nitrate leaching losses consist of pure nitrogen (0.69–0.81 kg ha^−2^)^44^. However, Yang determined that long-term straw application caused nitrate to leach into deep soil layers^32^. The main reasons supporting this phenomenon may be the short growing season and asynchrony between the crop growth period and precipitation; nitrate that has accumulated during a long period can be easily leached, so this time scale should be considered during long-term planning.

### Crop yields

In the present study, straw application increased crop yields. Compared with those in the control treatment, the rice yields in the T1 and T2 treatments increased by 7.79% and 14.56% in 2010, by 8.17% and 10.35% in 2012, and by 9.45% and 9.26% in 2013, respectively; the mean rice yields in T1 and T2 were 9.24% and 10.37% higher than the mean yields in the control. The winter wheat yields in T1 and T2 increased by 10.11% and 11.51%, respectively, in 2011. Zhang reported that rice straw application increases rice yields and that, compared with a total application, SS application leads to stronger yield gains^54^. Zhong reported that TS and SS applications can increase barley yields by 16.68% and 12.28%, respectively (P < 0.05)^55^. Zeng reported that crop yields increased by 1.7–145.8% (mean of 15.17%) in response to straw application at a rate of 1,500–9,000 kg ha^−2^ (mean of 4,611 kg ha^−2^) in China^56^. Li analysed the effects of straw application rates on crop yields and reported that wheat and corn yields decreased to 470 kg ha^−2^ (7.16%, P > 0.05) and 60 kg ha^−2^ (0.91%, P > 0.05), respectively, by straw application at a rate of 3,000 kg ha^−2^; in addition, the wheat yield decreased by 262 kg ha^−2^, and the corn yield increased by 113 kg ha^−2^ in response to straw application at a rate of 6,000 kg ha^−2^ ^57^. In general, a reduction in yield usually occurs in dry years, especially in arid regions and in non-irrigated zones. Yield reductions seldom occur in irrigated zones, so long-term straw applications benefit crop yields.

### Extension trials of rice straw applications

In 2012, an extension trial was conducted near the initial trial plots. The trial consisted of 3 treatments: a CK and SS and TS applications. The area of each treatment was 1/15 ha^2^. Compared with that in the CK, the soil nitrate in the SS and TS decreased by 12.13% and 18.02%, respectively, in the 0–30 cm soil layer. The soil ammonium concentration increased by 48.24% in the SS and by 65.84% in the TS treatments, but the concentration was only approximately one-tenth of the nitrate concentration. In addition, ammonium leaching losses were rare. In 2013, a more large-scale extension trial was conducted in Qingtongxia, Ningxia, at a site located 30 km west of the initial plots. The test area was 2.3 ha^2^ (1.9 ha^2^ received straw applications, and 0.4 ha^2^ served as controls). Compared with the control, straw application reduced nitrate leaching losses by 15.44%, and the rice yield improved by 7.23%.

### Shallow groundwater

Leached nitrates lost in the 90 cm soil layer have a greater chance to infiltrate water because the groundwater level is only 120–200 cm high during the planting season. The amount of leakage water was approximately 100–120 cm in the rice fields each year during the study, and the nitrate leaching losses were 8–8.69 kg pure nitrogen ha^−2^ y^−1^. If the leakage water and leached nitrates enter the groundwater together, the nitrogen concentration in the leachate would be 0.67–0.87 mg L^−1^, which is almost equal to 2.96–3.85 mg L^−1^ of the nitrate leachate. The nitrate concentration exceeded 10 mg L^−1^ in some places, and it was highest during the planting season. Zhao demonstrated that nitrate and organic nitrogen concentrations in the groundwater ranged from 11.85–26.12 and 0.64–5.89 mg L^−1^, respectively, and Zhao demonstrated that nitrate and organic nitrogen concentrations in the groundwater ranged from 11.85–26.12 and 0.64–5.89 mg L^−1^, respectively^58^. Ju noted that greenhouse vegetable farming caused soil nitrate accumulations and leaching: the groundwater nitrate concentration was 9–274 mg L^−1^, and the soil nitrate concentration was 270–5038 kg ha^−2^ in the 0–90 cm soil layer in Huimin County, Shandong Province, where 99% of the groundwater had nitrate concentrations greater than 10 mg L^−1 ^^59^. Song reported that soil nitrate leaching losses from greenhouse vegetables reached 152–347 kg ha^−2^ in the 0–100 cm soil layer in Shouguang County, Shandong Province and that those losses were closely related to groundwater pollution^60^.

## Conclusion

Straw application can reduce soil nitrate leaching losses. Nitrate leaching losses decreased by 12.71% to 12.84% in the Ningxia irrigation zone, an area in which TS application is recommended first because of low the SOM and low soil nutrient levels. Straw application improved the SOM by 2.76% to 6.83% and the TN by 0.89–1.24 g kg^−1^ in the 0–30 cm layer. As such, nitrogen fertilizer application rates could be reduced in the long term, and this approach could also reduce soil nitrogen leaching. Straw application could also increase crop yields. There are 0.7 billion tons of crop straw produced annually in China. This material is equivalent to 3.5 million tons of fertilizer nitrogen, 8 million tons of fertilizer potash, and 0.8 million tons of fertilizer phosphate. However, the rate of straw application is less than 50%^61^. For these reasons, more attention should be paid to both additional testing and to educating producers about the benefits of straw application in China.
